# Organoids and AI-integrated models in ovarian cancer research: the future of personalized therapy

**DOI:** 10.3389/fonc.2026.1903061

**Published:** 2026-09-16

**Authors:** Anuradha Nikam, Gyamcho Tshering Bhutia, Sinjini Sarkar

**Affiliations:** 1 Department of Pharmacology, Shobhaben Pratapbhai Patel School of Pharmacy & Technology Management, SVKM’s NMIMS Deemed-to-be-University, Mumbai, India; 2 Amity Institute of Pharmacy, Amity University Kolkata, Newtown, West Bengal, India

**Keywords:** artificial intelligence, machine learning, ovarian cancer, patient-derived organoids, precision oncology

## Abstract

Ovarian cancer (OC) is the deadliest gynecological malignancy which continues to impose a significant challenge in the field of oncology research due to its rapid metastasis, inter and intra-tumor heterogeneity, and treatment resistance. Personalized medicine for OC is still under development where patient-derived organoids (PDO) represent as a promising platform intended towards patient-specific disease modelling, biomarker discovery and therapeutic response assessment. These PDOs generate elaborate, complicated and high dimensional data traversing imaging, advanced omics and pharmacological analyses. Artificial intelligence (AI) and machine learning (ML) approaches allow refined prognostic stratification of patients through improved target delineation and response prediction when integrated with bioinformatics and conventional statistics. This narrative review discusses the foundations laid on the organoid generation and AI-enabled evaluation of ovarian cancer PDOs. The successful translation of AI-guided strategies into clinical oncology will depend on rigorous multi-center validation, harmonized methodologies, and prospective clinical studies demonstrating improvements in patient outcomes and real-world applicability.

## 1 Introduction

Ovarian cancer (OC) remains the second leading cause of gynecological cancer-related mortality with 206,956 deaths per year worldwide and a mortality-to-incidence ratio exceeding 0.6. Globally, it is the 18^th^ most common cancers with 324,603 incidences annually (1, 2). The high-grade serous ovarian cancer (HGSOC) is the predominant epithelial subtype, accounting for approximately 70–75% of cases and is characterized by near-universal *TP53* mutations together with frequent alterations in homologous recombination (HR) repair pathways, including *PTEN, BRCA1*, and *BRCA2* (3). Owing to the absence of specific early clinical symptoms, HGSOC is most often diagnosed at advanced stages, when extensive intraperitoneal dissemination has already occurred (4). Current clinical management relies on debulking surgery followed by platinum-based chemotherapy, most commonly in combination with taxanes (5). Although this therapeutic strategy achieves high initial response rates, most patients eventually relapse and develop chemoresistant disease, leading to poor long-term survival outcomes (6). Defects in HR repair define a clinically important subset of tumors that exhibit increased sensitivity to platinum-based chemotherapy and poly (ADP-ribose) polymerase inhibitors (PARPis). Although PARP inhibitors have improved progression-free survival (PFS) and are now incorporated into treatment strategies based on molecular testing for BRCA1/2 mutations, loss of heterozygosity, and HR status, patient stratification remains suboptimal, particularly in recurrent and drug-resistant disease, and consistent overall survival (OS) benefit remains limited (7).

Despite therapeutic refinements achieved through the novel formulations and targeted therapies, like pegylated liposomal doxorubicin and bevacizumab, OS has not improved substantially, indicating clear limitations of existing treatment strategies (8). Therapeutic failures are associated with pronounced intra-tumoral heterogeneity and continuous spatial and temporal genomic evolution under selective pressure from medical treatments (9). Such heterogeneity enables the coexistence of treatment-sensitive and treatment-resistant subclones within the same tumor, thereby limiting the predictive value of uniform therapeutic strategies. In addition to tumor cell-intrinsic mechanisms, the ovarian cancer microenvironment critically influences disease progression and therapeutic response. Malignant ascites establishes a tumor-supportive niche enriched with cytokines, growth factors, extracellular vesicles, and immune cells that promote tumor survival, dissemination, and immune evasion (10). Furthermore, interactions between cancer cells and stromal fibroblasts, immune infiltrates, and extracellular matrix components modulate drug sensitivity and contribute to adaptive resistance mechanisms (11). These complex and dynamic tumor microenvironment interactions remain inadequately captured by conventional experimental models and highlights the need for development of pre-clinical modeling to customize treatment.

The progress of personalized medicine is still in its infancy especially for the management of advanced ovarian cancer. A few biomarkers like folic acid receptor alpha (FOLR1), epidermal growth factor receptor 2 (HER2), and trophoblast cell surface antigen 2 (TROP2) were identified for antibody drug conjugates (12). Despite recent developments there are still a major requirement of biomarker driven patient stratification and customization of management of ovarian cancer especially for advanced and recurrent cases.

Patient-derived organoids have emerged as advanced three-dimensional (3D) culture systems that retain key structural, genetic, and functional properties of primary tumors. In ovarian cancer, organoids generated from surgical specimens and malignant ascites have demonstrated the ability to preserve tumor heterogeneity and recapitulate patient-specific drug responses *in vitro* (13). These models provide a scalable and experimentally tractable platform for functional drug testing and mechanistic studies. At the same time, organoid-based systems generate large, complex datasets encompassing morphology, growth dynamics, drug sensitivity profiles, and multi-omics information, presenting analytical challenges that exceed the capacity of conventional approaches (14). Here, AI is expected to complement the challenges by enabling automation in image analyses, advanced omics data analyses, and high throughput drug screening which results in identification of novel biomarkers and improved patient stratification. Thus, AI does not replace organoid technology; rather, it enhances its analytical capability, scalability, and potential for clinical translation toward functional precision medicine (**Figure 1**). Accordingly, this review critically evaluates the limitations of existing preclinical models, outlines the biological foundations of ovarian cancer organoids, and examines how their integration with AI-driven analytical approaches may inform future advances in precision oncology.

**Figure 1:**
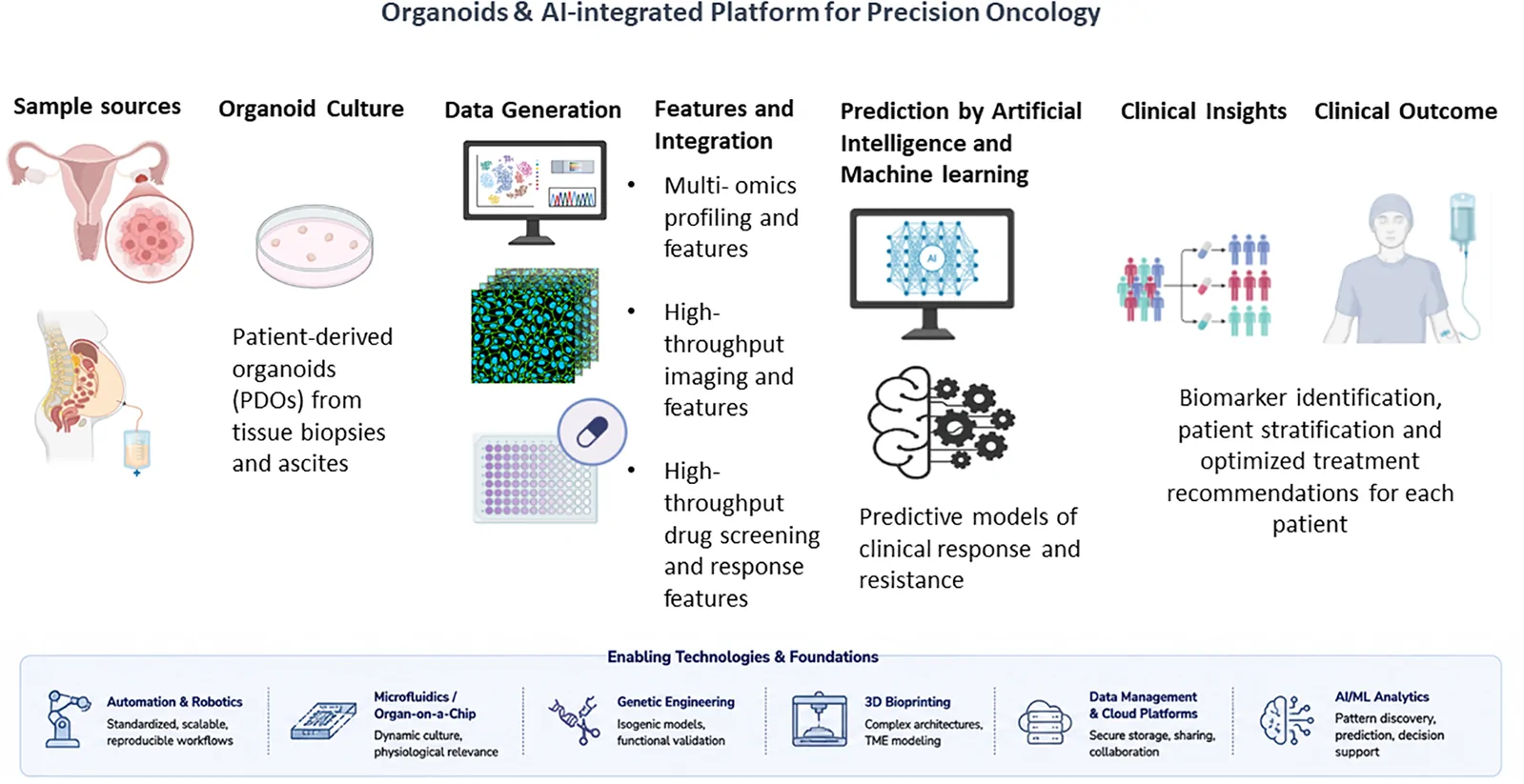
Representative study designs and translational applications of AI-integrated patient-derived organoid (PDO) platforms in ovarian cancer precision medicine.

## 2 Limitations of current preclinical platforms

2D cancer cell lines have historically formed the backbone of experimental cancer research owing to their technical simplicity, reproducibility, and suitability for high-throughput drug screening. These models have contributed substantially to elucidating oncogenic signaling pathways and basic mechanisms of drug action. However, prolonged *in vitro* culture induces genetic and epigenetic drift, promotes clonal selection, and leads to progressive loss of intratumoral heterogeneity, thereby diminishing their ability to reflect patient tumor complexity (15). In addition, growth on rigid plastic substrates imposes non-physiological mechanical cues that alter cell polarity, signaling networks, and metabolic programs altered energy metabolism, including reduced mitochondrial activity and disrupted biosynthetic and redox pathways, resulting in artificial cellular states that do not recapitulate *in vivo* tumor biology (16, 17). PDX (patient-derived xenograft) models have contributed to the study of tumor growth dynamics, metastatic behavior, and mechanisms of therapeutic resistance and were initially regarded as more biologically faithful than conventional 2D cell line systems (17). Nevertheless, PDX establishment in immunocompromised mice and expansion require high costs, while prolonged timeframes render them incompatible with clinical decision-making timelines, low throughput, and substantial animal-to-animal variability restrict their scalability for systematic drug screening (18). In addition, serial passaging results in progressive replacement of human stromal and immune components with murine counterparts, leading to limited utility of PDXs for evaluating immunotherapies, angiogenesis-targeted agents, and microenvironment-modulating strategies (19).

Genetically engineered murine models (GEMMs) have been instrumental in elucidating the functional roles of key genetic drivers, including TP53 loss and defects in DNA damage response pathways, and have provided important mechanistic insight into early tumorigenic events and disease pathogenesis (20). However, to their reliance on a limited set of engineered genetic alterations, these models fail to recapitulate the extensive genomic instability, clonal evolution, and inter-patient heterogeneity characteristic to human ovarian cancers (21). Moreover, species-specific differences in immune regulation, tumor–microenvironment interactions, and drug metabolism further constrain their predictive relevance (22). 3D tumor culture models were developed to address fundamental biological limitations of 2D systems, by permitting growth in all spatial dimensions, 3D models partially restore tissue-like architecture, biochemical gradients, and microenvironmental cues that regulate tumor behavior and therapeutic response (23). Yet, the absence of native vascular, immune, and stromal compartments limits the capacity of spheroids to model long-term tumor evolution and complex microenvironment-driven resistance mechanisms. Tumor explant models, while biologically faithful, are constrained by short viability windows, limited availability of patient material, and poor scalability, rendering them unsuitable for longitudinal studies or high-throughput drug screening (24). Despite substantial advances in the molecular and genomic characterization of ovarian cancer, progress in management of advanced cases remain inadequate largely due to the inadequate predictive capacity of existing preclinical models (25). These limitations have driven the development of patient-derived organoids as advanced platforms intended to bridge the gap between experimental modeling and clinical translation.

## 3 Patient-derived organoids: clinical relevance

Organoids are cellular *in vitro* models, which can reproduce the complex tumor stromal interaction and function as the corresponding *in vivo* organ/tissue. They can be engineered and dissected for fundamental mechanistic investigations on pathological progression, drug screening and personalized treatment. Organoids are derived from either pluripotent or tissue-resident stem (embryonic or adult)/progenitor/differentiated cells from healthy or diseased tissues, such as tumors. Malignant cell suspension is prepared by mechanical and enzymatic digestion followed by plating using basement membrane extract and supplemented with suitable medium. Organoid formation is dependent on embedding tumor cells within an extracellular matrix that provides structural support and biomechanical cues resembling the basement membrane, thereby enabling 3D organization and epithelial polarization (26).

## 4 Ovarian cancer organoids

### 4.1 Growth, histological subtypes and molecular characterizations

Primary tumors resected from the malignant ovary or fallopian tube, metastatic lesions from peritoneal or omental sites, and malignant ascites collected from patients with advanced-stage (II, III and IV) disease serve as main tissue source for organoid development (27). In recent times, fluid- derived organoids or FDOs are also being developed from cells isolated from pleural effusion of lung and ovarian cancers (28). Transcriptomic profiling from the malignant effusions of platinum resistant serous ovarian carcinoma uncovered several targets (29). However, protocols for standardization of FDOs are still evolving for routine clinical decision-making (28). To complement primary tissue modeling, established human ovarian adenocarcinoma lines including SKOV-3, OVCAR-3, IGROV1, and A2780 have also been successfully adapted to generate 3D organoid and spheroid architectures (30–32).

Morphologically, ovarian cancer organoids arise from either single isolated tumor cells or micro-clusters, achieving self-organized, mature 3D structures within 1 to 2 weeks post-seeding. The growth kinetics of these cultures demonstrate a strong dependence on initial seeding density. For high-throughput screenability and biobanking, long-term expansion potential is critical. Optimized ovarian organoid lines demonstrate high passage stability, maintaining viable long-term culture up to 30 passages without significant loss of self-renewal capacity (21, 33). Furthermore, these platforms exhibit robust biobanking fidelity, with reported post-thaw recovery rates of approximately 85% following standard cryopreservation protocols (33). In addition, the slow growth of OC organoids were improved by reducing Wnt activity and active BMP (bone morphogenetic protein) signaling in HGSOC samples which mimicked the patient’s tumor characteristics in a breakthrough study having potential for personalized therapy (34). Another study reported Neuregulin-1 (NRG) as a crucial growth factor for HGSOC derived organoids which maintains the epithelial lineage long-term (35).

Several other studies have also reported preservation of patient-specific mutational landscapes like ubiquitous TP53 alterations, variable defects in HR repair pathways, PARPi sensitivity, retention of intra-patient heterogeneity and which establishes PDOs as genetically faithful representations of the original tumor (36). This structural preservation provides a biologically relevant 3D framework for modeling tumor behavior *in vitro*. Alves-Vale et al. (37) demonstrated close histological concordance between patient-derived tubo-ovarian carcinoma organoids and matched parental tumors. Their results were supported by comparative H&E, morphology and concordant immunohistochemical expression of Müllerian and epithelial markers, including PAX8 (paired box 8), CK7 ( Cytokeratin 7), and tumor-specific p53 staining patterns (37). PDO biobanks enable high-throughput screening of chemotherapy and targeted agents across genetically diverse tumors. The **Table 1** summarizes about the histological types represented in existing PDO studies, the culture methods used, and the longest reported passage times.

**Table 1:** Description of different ovarian cancer PDOs from separate histological subtypes.

Histological subtype	Representative studies (PDO series/biobanks)	Typical culture matrix & media (key factors)	Longest reported passage/duration	Main applications
High-grade serous (HGSOC)	Kopper et al. Nat Med 2019 (21); de Witte et al. Cell Rep Med 2020 (38); Hoffmann et al. (34); Vias et al. eLife 2023 (39); Nanki et al. Sci Rep 2020 (40). *Most common*(often >50% of lines)	Matrigel/BME or collagen-based 3D embed; advanced DMEM/F12 with Wnt3a, R-spondin, EGF, FGF(2/10), Noggin, N-acetylcysteine, Gastrin, NRG1, etc	>20–30 passages(many lines sustained >10 months)	Drug screening (platinum, PARPi, targeted agents); biomarker discovery; modeling chemo-resistance and intratumor heterogeneity
Endometrioid	Kopper et al. Nat Med 2019 (21); Yoshiko et al. Sci Rep 2020 (41); Maru et al., 2019 (42). *Moderately represented*	Matrigel/BME with similar niche factors (Wnt activators, EGF, FGF, etc.)	Often 5–15 passages (some lines passaged for months)	Molecular profiling; targeted drug studies (e.g. PI3K/Akt pathway); modeling ARID1A-mutant cancers
Clear cell (OCCC)	Kopper et al.2019 (21); Yoshiko et al., 2020 (41); Hill et al.2018 (33); Maenhoudt et al., 2020 (35). *Moderately represented*	Matrigel/BME with Wnt/RSPO, EGF, FGF, etc. (similar to above)	Typically 2–10 passages(maintained for weeks to few months)	Studying chemoresistance (OCCC is platinum-resistant); targeted therapy (e.g. ARID1A-mutated models)
Mucinous (MOC)	Kopper et al.2019 (21); Hippo et al. (42); Yoshiko et al.2020 (mucinous borderline) (41). *Rare* (few lines reported)	Matrigel/BME; some groups use modified protocols (Matrigel bilayer organoid culture); niche factors including EGF, FGF, RSPO	Limited data (often short-term cultures)	Disease modeling of mucinous tumors; potential drug testing (rare, not routine)
Low-grade serous (LGSC)	Kopper et al.2019 (21); Hippo et al.reports (42). *Rare*	Matrigel/BME with EGF, FGF, Noggin, Wnt factors	Limited data (few lines, short culture durations)	Functional studies; hormone response modeling (LGSC is often ER-positive)
Borderline & others (e.g. SBT, Brenner)	Hippo et al. (42) (MBOC protocol) (9 lines including serous borderline, Brenner, early-stage). *Rare*	Matrigel/BME (often modified bilayer protocol)	Variable; in one study 9 lines achieved 60–83% efficiency	

PDO, patient-derived organoid; HGSOC, high-grade serous ovarian cancer; OCCC, ovarian clear cell carcinoma; MOC, mucinous ovarian carcinoma; LGSC, low-grade serous ovarian carcinoma; SBT, serous borderline tumor; BME, basement membrane extract; DMEM/F12, Dulbecco’s Modified Eagle Medium/Nutrient Mixture F-12; Wnt3a, Wingless-related integration site 3a; RSPO, R-spondin; EGF, epidermal growth factor; FGF, fibroblast growth factor; NRG1, neuregulin 1; PARPi, poly(ADP-ribose) polymerase inhibitor; PI3K/Akt, phosphoinositide 3-kinase/protein kinase B; ARID1A, AT-rich interaction domain 1A; ER, estrogen receptor; MBOC, Matrigel bilayer organoid culture.

### 4.2 Drug screening, drug response and resistance

Organoids typically take a few weeks to grow, making them useful during critical periods when treatment decisions for patients are made. Studies have demonstrated that ovarian cancer organoids reproduce relative treatment sensitivities observed in patients, enabling functional stratification beyond genomic profiling alone. Organoids derived from homologous recombination-deficient (HRD) tumors consistently exhibit enhanced sensitivity to platinum agents and PARP inhibitors, whereas non-HRD organoids display reduced responsiveness, mirroring clinically observed patterns of therapeutic benefit (36). Epithelial ovarian carcinoma derived organoids predicted response to first-line treatment i.e. Carboplatin/Paclitaxel and PARP inhibitors (43). Chen et al., 2024 (44) describes *in vitro* drug testing on 10 PDOs from advanced OC patients using guideline- recommended drugs like taxanes, platinums, PARPis, epirubucin, doxorubicin, gemcitabine and topotecan. They report that the results of organoid drug testing were consistent with their parent tumors and determined the course of therapy for the patients. They were categorized as platinum sensitive, resistant and refractory cases (44). Another research extensively studied 36 whole-genome sequenced OC PDOs were for chemotherapy sensitivities while it also reports that similar genomic traits remained in the PDOs cultured. As a pilot experiment, they performed the evaluation of drug responses within a span of 20 days from tumor isolation from recurrent OC cases (38). They were able to investigate and identify at least one drug with high sensitivity among 88% of the patients. In another study, ovarian cancer PDOs demonstrated excellent clinical predictive performance, showing complete agreement with prior patient treatment responses and 80% accuracy in prospective therapeutic prediction. Furthermore, preoperative serum CA15–3 and CA19–9 levels were identified as independent factors associated with successful PDO generation (45).

In addition, organoids established from recurrent or treatment-exposed tumors frequently recapitulate chemoresistant phenotypes, providing an experimentally tractable system for investigating the emergence and persistence of acquired resistance under therapeutic pressure (46). Keratin 17 (KRT17) had been associated with platinum resistance and a negative prognostic marker, and upregulation of KRT17 in organoids demonstrated increased sensitivity towards Alpelisib and Afuresertib, making it a potential marker for patient stratification with PI3K/Akt pathway targeted treatment (47). (36) have extensively reviewed about the role of PDOs in determining PARP sensitivity and resistance with next generation sequencing (NGS) based biomarker identifications (36).

## 5 Bioinformatic preprocessing and conventional statistics

Patient-derived organoids (PDOs) generate multi-omics and imaging data, through high- throughput sequencing approaches and label-free microscopy (27, 48). These datasets are initially processed by bioinformatics approaches. For example, Harmony and ComBat are two leading bioinformatics algorithms used on raw sequencing data for batch effect correction and dimensionality reduction in genomic and transcriptomic data integration. Single-cell RNA sequencing (scRNA-seq) data are analyzed using bioinformatics frameworks such as Seurat for cell clustering and annotation. Following which, Gene Set Enrichment Analysis (GSEA) and Over-Representation Analysis (ORA) are employed to identify predefined gene sets are enriched and significantly associated biological pathways. AUCell is then applied to map the pathway activity back to individual cells and stratify subsequent cellular populations. In addition, live-cell imaging enables real-time monitoring of organoid growth, invasion, and morphological changes without the need for staining techniques (49). Prognostic genes are commonly screened using univariate cox proportional hazards regression relying heavily on *p-values*. Artificial intelligence approaches have expanded the analytical capabilities of cancer research beyond these classical methods.

## 6 Artificial intelligence-integrated ovarian cancer organoids

Artificial Intelligence (AI) can interpret the vast and complex datasets produced by modern biological research using machine learning (ML), deep learning (DL), and natural language processing (NLP). Deep learning approaches, such as Light Gradient Boosting Machine (LightGBM) and convolutional neural networks (CNNs) integrate drug fingerprint data with the molecular characteristics of PDOs to predict therapeutic responses (50, 51). ML algorithms learn from training data to classify, cluster, or predict outcomes in unseen datasets, uncovering complex patterns beyond conventional analytical methods while enabling standardized, reproducible, and scalable data analysis (52).

Ovarian cancer organoid droplets’ growth prediction model was developed by feeding 517 longitudinal microscopy images to four neural network (ResNet18, VGG11, ConvNeXt v2, and Swin Transformer v2) backbones which were then trained and validated. The developed model achieved satisfactory results with improved AUC 0.884 (P = 0.0039) (53). By integrating multimodal omics profiles with longitudinal treatment data, an AI model predicted platinum-sensitive and platinum-resistant recurrence following therapy. ALDH1A1(Aldehyde dehydrogenase 1 family member A1)- associated gene signatures identified prognostic biomarkers for chemotherapy response and guided AI-assisted high-throughput screening of potential inhibitors, which were subsequently validated in cell lines and ovarian cancer organoids (54).

## 7 Limitations and scope in precision oncology

High-grade serous ovarian cancer (HGSOC) makes up to ~70% of epithelial ovarian cancers and is the main cause of chemoresistance-related mortality. Apart from a few established protein markers, polymorphisms and miRNA expressions, clinical biomarkers are scarce which predict response to platinum-based chemotherapy in ovarian cancer (55–57). Precision oncology initiatives have largely focused on genomic and transcriptomic profiling to guide treatment selection. Despite their biological relevance, a principal constraint in PDOs is the absence of native immune, vascular, and stromal components, which restricts the ability of standard organoid cultures to fully recapitulate tumor–microenvironment interactions that critically influence disease progression and therapeutic response (58). In addition, organoid establishment relies on extracellular matrix scaffolds that introduce batch-to-batch variability and non-physiological biochemical cues, potentially affecting growth dynamics and while culture conditions also tend to favor epithelial tumor populations, which may lead to underrepresentation of mesenchymal or rare cellular states present within the original tumor (59). In addition, studies with AI integration especially focused on ovarian cancer PDOs are still scarce and yet to have advanced to clinical validation.

Human trials incorporating organoid-based predictions are nascent. AI models also need external multi-center data to prove generalizability. Larger multi-center biobanks, federated learning, and explainable AI frameworks are needed to address these issues. To summarize, the key challenges include data size and heterogeneity (PDO datasets are small relative to cell line collections, making deep learning difficult); standardization (culture conditions, imaging hardware and annotation varies across centers); interpretability (black-box models erode trust and the identification of causal biomarkers); reproducibility (risk of overfitting necessitates rigorous cross-validation and open benchmarking); and regulatory/ethical issues (patient-derived data privacy, AI transparency and regulatory approval of AI diagnostics) (60). While ongoing efforts aim to address these issues, such limitations underscore the need for cautious interpretation of organoid-based findings and motivate continued methodological refinement.

However, a few promising results show hope towards personalization of treatment in OC. A novel HGSOC organoid system was derived from patient samples which was able to sustain critical immune microenvironment and blood vessels. The system was utilized to assess cisplatin sensitivity (61). Neural network models trained on organoid brightfield images and genomic profiles can identify gene signatures associated with drug resistance. Imaging-based ML (e.g. CNNs) has achieved high segmentation accuracy (Dice~0.91) and can classify organoid viability (~78% concordance with experts) faster than dyes (49, 62). Multi-omics and drug fingerprint integration in AI-driven pipelines show high predictive power (e.g. PR AUC≈0.81 in >100 PDO lines). These integrated PDO-AI platforms have been validated *in vitro* and *in vivo* (xenografts) and show promise to identify biomarkers (e.g. KRT17, ALDH1A1) and drug synergies for stratified therapy (21). Challenges remain in data standardization, external validation (e.g. ovarian PDO viability models AUC dropped from 0.86 to 0.63 on independent data), interpretability and clinical translation (63, 64). Some suggested study designs with expected outcomes are given below in **Table 2**.

**Table 2:** Representative study designs and translational applications of artificial intelligence-integrated patient-derived organoid (PDO) platforms in ovarian cancer precision medicine.

Study design	Sample size	Endpoints	Expected deliverables
Prospective PDO Drug Screen	50–100 patients	PDO IC50 vs. clinical response; safety of culture	Predictive model for chemotherapy sensitivity; validity of PDO platform as predictive assay
Imaging–AI Assay Development	10–20 PDO lines	Segmentation accuracy (Dice/F1); viability classification accuracy vs. dye	Validated CNN for organoid segmentation; ML classifier (accuracy ~80%+) for live/dead status
Multi-omics Profiling of PDOs	20–30 PDO lines	Correlation of genomics/proteomics with drug response (AUC, C-index)	Integrated ML model (e.g. random forest) linking omics features to drug sensitivity; biomarker panels
Integrated PDO–Patient Cohort	30–50 PDO–patient pairs	Concordance (ROC/AUC) between PDO predictions and patient outcomes	Clinical-grade prediction nomogram combining AI score + clinical factors (e.g. AUC>0.8)
Drug Combination Discovery	5 PDO lines + *in vivo*	Synergy metrics (Bliss/ZIP), tumor growth inhibition in mice	Candidate synergistic regimens validated in organoids and xenografts; mechanistic hypotheses
AI Model Generalizability Test	External PDO cohort (20)	Model AUC, PR-AUC on independent data	Calibration report and refined model after transfer learning; guidelines for cross-center use

Endpoints include metrics like AUC, C-index for survival, Dice/F1 for imaging segmentation, PR-AUC for classifiers.PDO, patient-derived organoid; IC₅₀, half-maximal inhibitory concentration; CNN, convolutional neural network; ML, machine learning; F1, F1 score; AUC, area under the curve; C-index, concordance index; ROC, receiver operating characteristic; AI, artificial intelligence; ZIP, zero interaction potency; PR-AUC, precision–recall area under the curve; Dice, Dice similarity coefficient.

The integration of immune and stromal co-culture systems, microfluidic technologies, single-cell metabolomics, clinical metabolomic datasets, and AI/ML approaches can substantially enhance the physiological relevance, analytical capability, and predictive performance of organoid models. AI-guided mechanistic mathematical models enable *in silico* study modifications whereas, AI-guided robotics streamlines experimental validation, which accelerates organoid research (65). The convergence of high-throughput organoid platforms with organ-on-a-chip technologies, genetic engineering, three-dimensional (3D) bioprinting, and AI-driven analytical frameworks holds considerable promise for accelerating translational ovarian cancer research and advancing functional precision medicine.

## 8 Conclusion

In terms of disease modelling, prognosis, treatment and resistance development, organoids empowered with AI present interesting paths for the advancement of precision medicine in OC. Nevertheless, most of the evidence now available is exploratory, and enhanced statistical performance has not yet resulted in proven clinical benefit. Before these methods may be incorporated into standard practice, thorough prospective validation and decision-impact studies are necessary.
